# Immune-depleted tumor microenvironment is associated with poor outcomes and BTK inhibitor resistance in mantle cell lymphoma

**DOI:** 10.1038/s41408-023-00927-2

**Published:** 2023-10-12

**Authors:** Preetesh Jain, Krystle Nomie, Nikita Kotlov, VitaIy Segodin, Holly Hill, Chi Young Ok, Ahmed Fetooh, Rashmi Kanagal-Shamanna, Francisco Vega, Alexander Bagaev, Nathan Fowler, Christopher R. Flowers, Michael Wang

**Affiliations:** 1 Department of Lymphoma and Myeloma from The University of Texas MD Anderson Cancer Center, Houston, TX USA; 2 BostonGene Corporation, Boston, MA USA; 3 Department of Hematopathology at The University of Texas MD Anderson Cancer Center, Houston, TX USA

**Keywords:** Lymphoma, Immunopathogenesis

Mantle cell lymphoma (MCL) is a generally aggressive B cell non-Hodgkin lymphoma (B-NHL). Outcomes of patients have improved in the era of Bruton’s tyrosine kinase inhibitors (BTKi). However, MCL patients can develop resistance to BTKi over time and can progress [1]. Ibrutinib resistance in MCL is correlated with an overexpression of OXPHOS, MYC and PI3K/AKT/m-TOR pathways. Somatic mutations in *TP53, KMT2D, NSD2, SMARCA4, CCND1, TRAF2, NFKBIE* genes are reported with ibrutinib resistance [2]. A few reports have also demonstrated that a decrease in T cell numbers [3, 4] or a downregulation of effector/cytotoxic T cells [5, 6], high Tregs [7] or a decreased expression of T cell activation/co-stimulation pathways [5] are associated with resistant MCL. An understanding of the tumor microenvironment (TME) and its cellular and analyte composition plays a critical role in promoting MCL cell growth, proliferation [8] and treatment resistance [6]. Unlike other lymphomas [9], the TME in MCL patients has not been fully characterized at the transcriptomic and genomic levels. To further understand the relevance of the tumor-immune landscape in tissue microenvironments, we performed multiomic profiling to characterize the TME in tissues from MCL patients and examined the relationship between TME subtypes and their impact on clinical outcome and the response to BTKi.

This cohort study was conducted under an IRB approved protocol for MCL patients at our Center. Tissue biopsies (30 lymph nodes and 11 other tissues) were collected from 41 patients with MCL (patient characteristics in supplemental Table 1). Samples were obtained at progression in patients who were resistant or before starting treatment with BTKi in patients who were sensitive to BTKi. Primary BTKi resistant MCL were patients who never responded while acquired resistant MCL were those who had either a partial or complete response to BTKi and subsequently progressed. Among evaluable patients, DNA and RNA extraction was performed from fresh biopsies from lymph nodes and non-nodal tissues. Whole exome (WES) and bulk RNA sequencing were performed to assess the somatic mutation profile, copy number abnormalities and gene expression profile to identify TME gene clusters. RNA sequencing data were combined with data from an independent cohort of MCL patients [Scott et al. (*n* = 122)] [10]. Fig. 1A displays the study design. Joint WES and RNA-seq mutation calling, expression analysis, and cell deconvolution from the transcriptome were performed using the BostonGene automated pipeline [9]. All WES and bulk RNA sequencing was performed with Illumina HiSeq4000 using a 76 bp paired end configuration (described in the supplemental file). Overall survival was calculated from the initiation of BTKi therapy until death or the date of last follow up (censored).

**Fig. 1:**
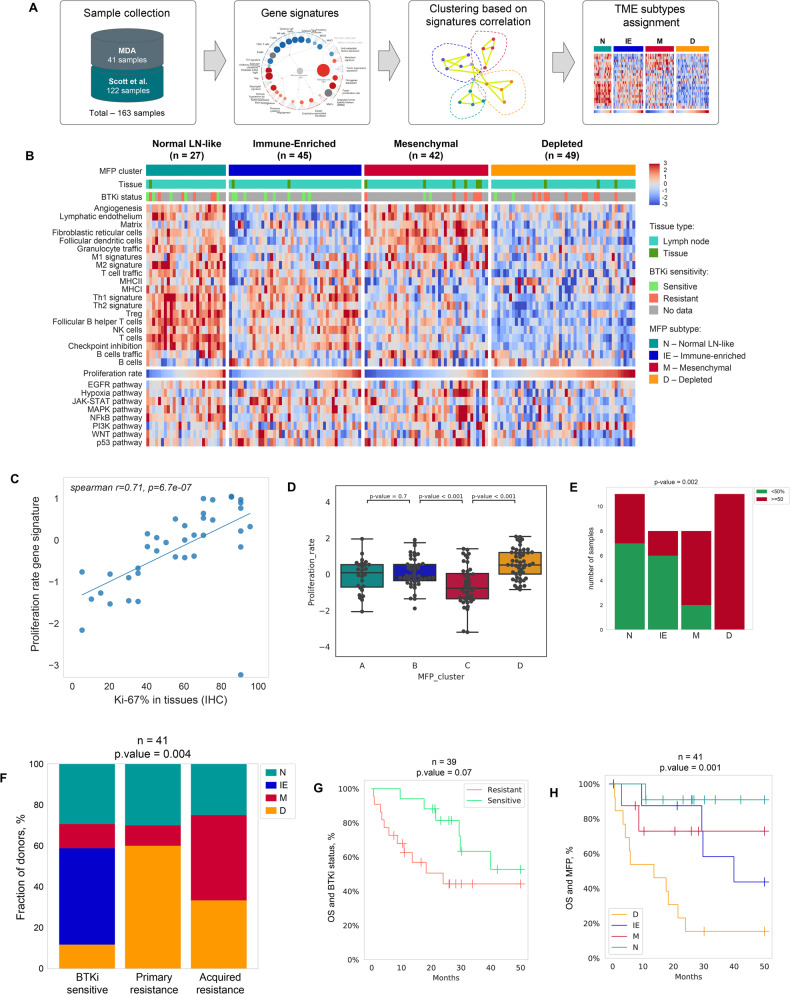
Study design, transcriptomic profile showing tumor microenvironment (TME) clusters in mantle cell lymphoma tissue biopsies, their response to ibrutinib and impact on survival outcomes. **A** Schematic representation of the Molecular Functional Portrait (MFP) discovery and TME clusters from 41 patient samples from MD Anderson and 122 patient samples from previous analysis by Scott et al. [10]. **B** Unsupervised clustering based on activities of proposed signatures identified four MFP clusters, as shown in a heatmap with gene signature values (including a proliferation rate signature). Each column represents an individual patient sample. PROGENY pathways are shown below proliferation rate signature. Red values are high gene expression, blues are low gene expression. The abbreviations for the four clusters are N normal lymph *node*like, IE immune-enriched, M mesenchymal and D depleted “Immune-cold”. Tissue type (lymph node vs non-nodal tissue) and clinical response to BTK inhibitor are depicted in the legend. **C** Scatter plot showing a significant correlation of Ki-67% in tissue biopsies with the gene proliferation rate signature by bulk RNA sequencing in BTKi treated patients at MD Anderson (*n* = 41; spearman correlation coefficient r2-0.71). Genes from tumor proliferation rate signature include: *CCND1, E2F1, ESCO2, MKI67, MYBL2, CETN3, CDK2, BUB1, CCNB1, CCNE1, MCM6, AURKB, MCM2, PLK1, AURKA.* **D** Box plots demonstrating the relationship of proliferation rate signature with MFP clusters. Immune depleted subtype had the highest proliferation rate (*P* < 0.001). **E** Ki-67% by immunohistochemistry (IHC) and MFP clusters showing that immune depleted subtype had the highest Ki-67% by IHC proliferation rate (*P* = 0.002). **F** Pattern of distribution of TME clusters (%) and the number of patients according to the response to BTK inhibitors (sensitive vs acquired and primary resistant patients). Immune-depleted or immune-cold TME cluster is seen in 50% patients with primary BTKi resistance (*P* = 0.004). **G** Kaplan–Meier overall survival (OS) curve according to response to BTKi shows that resistant patients were not significantly inferior in OS compared to those with BTKi sensitive disease (*p* = 0.07) inferior overall survival. **H** OS according to TME clusters shows a significantly inferior OS in immune depleted (D in orange) cluster as compared to mesenchymal (M in red), immune enriched (IE in blue) and normal lymph node like (N in green color) TME cluster, *p* = 0.001.

Unsupervised clustering identified four MCL TME subtypes reflecting distinct tumor-immune cell gene signatures. Figure 1B depicts the four distinct MCL TME subtypes—normal lymph node-like [9] (**N**; *n* = 27), immune cell-enriched or “Hot” (**IE**; *n* = 45), mesenchymal (**M**; *n* = 43) and immune-depleted or ‘Cold’ (**D**; *n* = 49). The LN-like subtype was enriched with components of the normal LN, “stroma-rich” especially CD4 + T-cells; follicular dendritic cells (FDC), T-follicular helper cells (TFH) cells, and lymphatic endothelium. Other TME groups were composed of overexpression of immune and checkpoint molecules with low stromal expression in (**IE**), non-immune with increased stromal signature and tumor-promoting cytokines (**M**) and immune-depleted (**D**) category possessed the highest content of malignant B cells. In the lower columns in Fig. 1B, we show that the tumor proliferation rate signature is significantly overexpressed in (**D**) TME group. Supplemental Fig. 1A shows that differences in tissue type did not influence the variability of TME among the tissue types.

Evaluable patients were further classified based on response to BTKi as sensitive (responders; *n* = 16), primary resistant (never responders and progressed on BTKi; *n* = 11) and acquired resistant (responded and later relapsed with progressive disease; *n* = 12). The majority of patients were treated with ibrutinib (*n* = 34), 6 acalabrutinib and one zanubrutinib.

TME was further dichotomized into immune cell rich and immune desert categories based on commonly involved immune cells and pathways (Supplemental Fig. 1B). Commonly affected pathways in MCL were evaluated based on TME subtypes (Supplemental Fig. 1C) and PI3K pathway genes were overexpressed, while JAK-STAT, MAPK and p53 genes were downregulated in **D** subtype. To evaluate if check point molecules were distributed according to the TME subtypes, we assessed the distribution of checkpoint molecules across TME subtypes, and noted that **D** and **M** subtypes had a lower expression of immune checkpoint genes compared with the IE TME subtype (Supplemental Fig. 2).

We further observed that Ki-67% from tissue biopsies had a linear correlation with proliferation rate signature genes [9] (Fig. 1C–E) and significantly overexpressed in the **D** group (*p* = 0.002) (box plots in Supplemental Fig. 3).

To explore the somatic mutation profile in relation to gene expression based TME clusters, we performed multiomic analysis combining WES data with RNA sequencing data depicted according to the four TME clusters (Fig. 2A, Supplemental Fig. 4A). Somatic mutations such as *TP53, NSD2, NOTCH1, KMT2D, SMARCA4*, which were previously reported in ibrutinib-resistant MCL and/or in refractory high-risk MCL patients, were predominant in the **D** subtype. Prominent mutations in **D** subtype and their location in each gene is shown in lollypop plots in Fig. 2B. We further described the somatic mutation data by the degree of ploidy, chromosomal instability, aneuploidy and copy number abnormalities in Supplemental Fig. 4. Tumor fraction was highest in the D subtype (*p* < 0.05). Subtypes **M** (red) and **D** (orange) had a relatively higher degree of chromosomal instability (*p* = n.s). Multiomic profiling identified mutations affecting the cell cycle, Notch, and chromatin regulatory pathways in TME cluster **D**. These pathways were seen in MCL tumors that were resistant to BTKi (Supplemental Fig. 5).

**Fig. 2:**
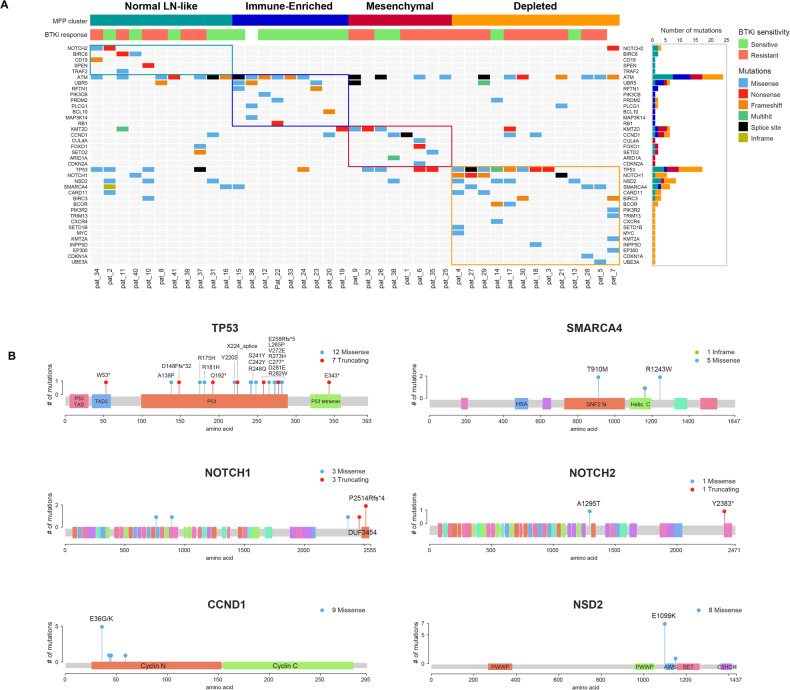
Somatic mutation profile by whole exome sequencing (WES) and bulk RNA sequencing and association with tumor microenvironment (TME) clusters in mantle cell lymphoma tissue biopsies. **A** Oncoplot showing distribution of somatic mutations with bar plots on the right side showing distribution of highly mutated genes in MCL according to the four TME clusters (shown on top of the oncoplot). Immune depleted (orange), mesenchymal (red), immune enriched (blue) and normal lymph node like TME cluster (green). *TP53*, *NOTCH1*, *NSD2*, *SMARCA4* mutations previously reported with clinical BTKi resistance in MCL were predominantly observed in immune-depleted TME cluster. **B** Somatic mutations in highly mutated genes in MCL. Protein domain structure of the 6 highly mutated genes with somatic mutations aligned, with positions and frequencies of the specific mutations is shown. Different colors as shown in the legend indicated the functional domains and mutation types in each gene—*TP53, NOTCH1, NSD2, SMARCA4, NOTCH2*, and *CCND1* genes.

Since our data demonstrated that TME subgroups separated MCL into four subtypes, we then evaluated the pattern of distribution of immunoglobulin heavy chain variable region genes (IGHV) gene usage according to the TME clusters and BTKi response status (Supplemental Fig. 6). VH3 gene usage was highest among the various VH genes but significant correlation of MFP clusters or BTKi response could not be identified.

Finally, the evaluable patients (*n* = 39) were divided according to response to BTKi- sensitive, primary resistant and acquired resistant and MFP clusters. Patients with primary and acquired resistance had significant proportion of patients with **D** subtype (*p* = 0.004; Fig. 1F), compared to N and IE subtypes. Primary and BTKi resistant patients had a trend of inferior survival compared to sensitive patients (*p* = 0.07; Fig. 1G). Furthermore, we demonstrated that the **D** TME group had worse overall survival compared to other TME categories (*p* = 0.001; Fig. 1H).

The role of TME and its components in mediating resistance to BTKi and in the propagation and maintenance of MCL cells remains under active investigation [11]. We described the molecular functional portrait (MFP) of MCL with four subtypes of MCL derived from multi-omic analysis. Immune-depleted gene expression subgroup has recently been reported [9] to indicate a poor prognosis in diffuse large B cell lymphoma (DLBCL) and exhibit a proliferative gene signature. An immune-depleted TME in lymphoma was shown to exhibit hypermethylation of SMAD promoter [12] leading to immune escape of lymphoma cells from microenvironmental influence and acquire *TP53* mutations in DLBCL. Furthermore, reduced expression of MHCI/II transcripts was observed in the **D** group, indicating an immune suppressive microenvironment which is associated with resistance. Recently, another study reported that SOX-11 positive MCL tissues [5] exhibit reduced T cell infiltration and lower MHC class I/II expression and a lower expression of T cell activation/co-stimulatory genes which are correlated with reduced OS [13].

We further demonstrated that Ki-67% by IHC is significantly correlated with the proliferation gene signature expression. The highest proliferation rate was noted in D subtype and almost all patients with high Ki-67% (>50%) had D subtype of MFP (Fig. 1C–E). At the same time T, NK cell and other immune signatures are very low for D subtype while enriched in tumor fraction. Furthermore, in **D** subtype, MCL patients exhibited high-risk somatic mutations, thereby exhibiting a significantly inferior OS compared to other subtypes. In addition, both primary and acquired BTKi resistant MCL patients exhibited a dominance of immune depleted signature. In addition, PD-L1 was significantly downregulated in the “**D”** MCL (*p* = 0.001) and PI3K was overexpressed, indicating that targeting the PD-L1 and PD-1 immune checkpoint axis may not appear beneficial in BTKi-resistant MCL, while PI3K pathway modulation could be a potential therapeutic target in these patients.

Our findings indicate a thought-provoking report on MFP clusters in MCL, showing that an immune depleted subtype is associated with poor outcomes and BTKi resistance in MCL. The study is limited by small number of patients and limited tissue availability for sequencing and a larger cohort of BTKi treated MCL patients in the frontline setting would be ideal to provide robust results. Our data suggests that the TME has a prominent role in mediating response to BTKi. The immunosuppressive TME in MCL requires further investigation to understand drug resistance. These multi-omic data provide a novel avenue to study the mechanics of the interactions of immune cell-tumor landscape and its alteration by BTKi in MCL patients.

## Supplementary information

Supplemental Figure-6

Supplemental Figure-1

Supplemental Figure-2

Supplemental Figure-3

Supplemental Figure-4

Supplemental Figure-5

Supplemental Table and figure legends
