# Supplementary figures and images for: Immune-depleted tumor microenvironment is associated with poor outcomes and BTK inhibitor resistance in mantle cell lymphoma
Source: Blood Cancer J. 2023 Oct 12;13(1):156. doi: 10.1038/s41408-023-00927-2 (PMC10567800; doi:10.1038/s41408-023-00927-2)

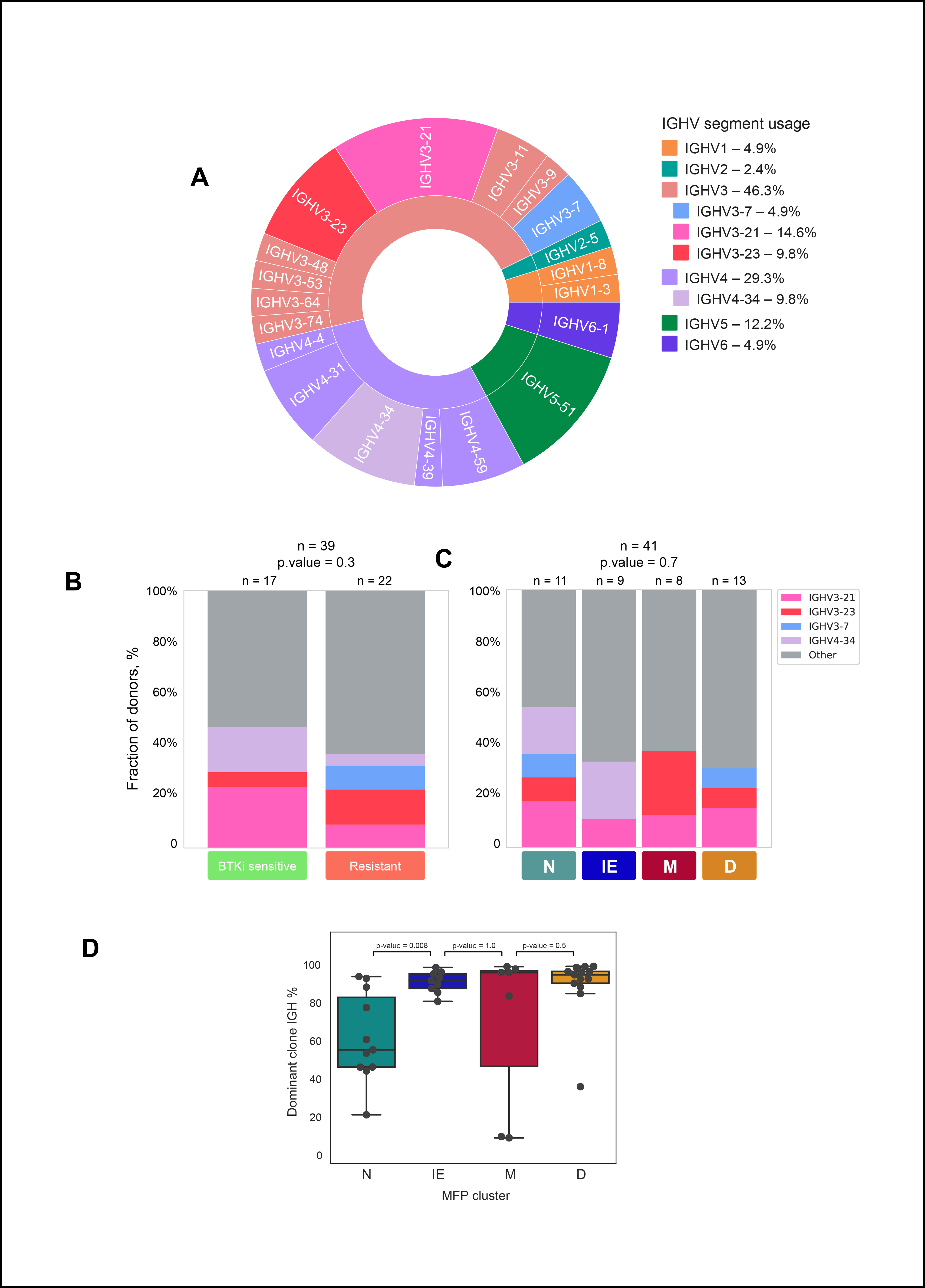

Supplement: Supplementary file 1 — Supplemental Figure-6 [file 41408_2023_927_MOESM1_ESM.png]

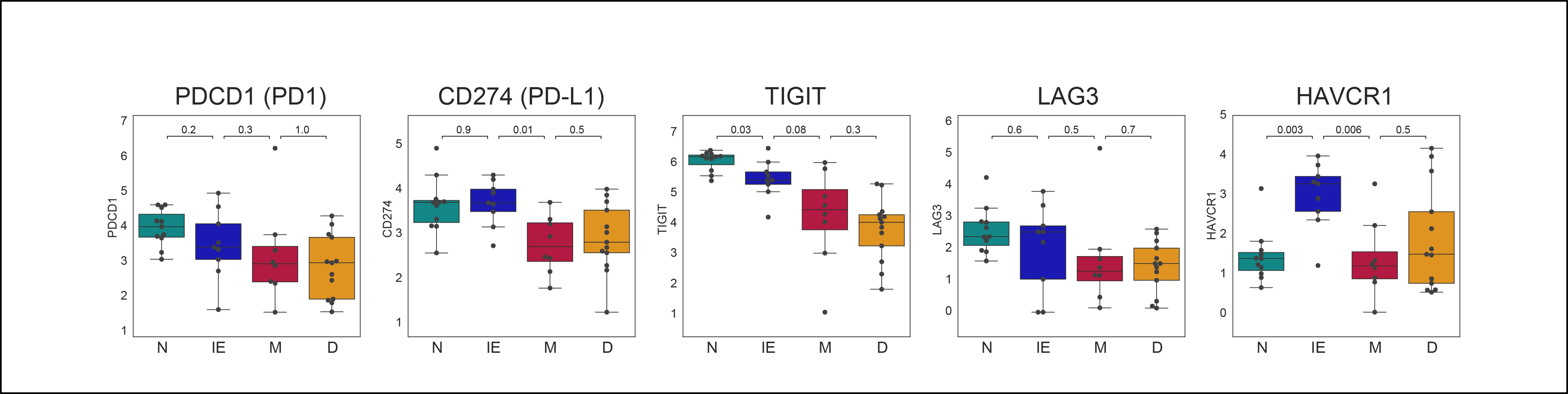

Supplement: Supplementary file 3 — Supplemental Figure-2 [file 41408_2023_927_MOESM3_ESM.tif]

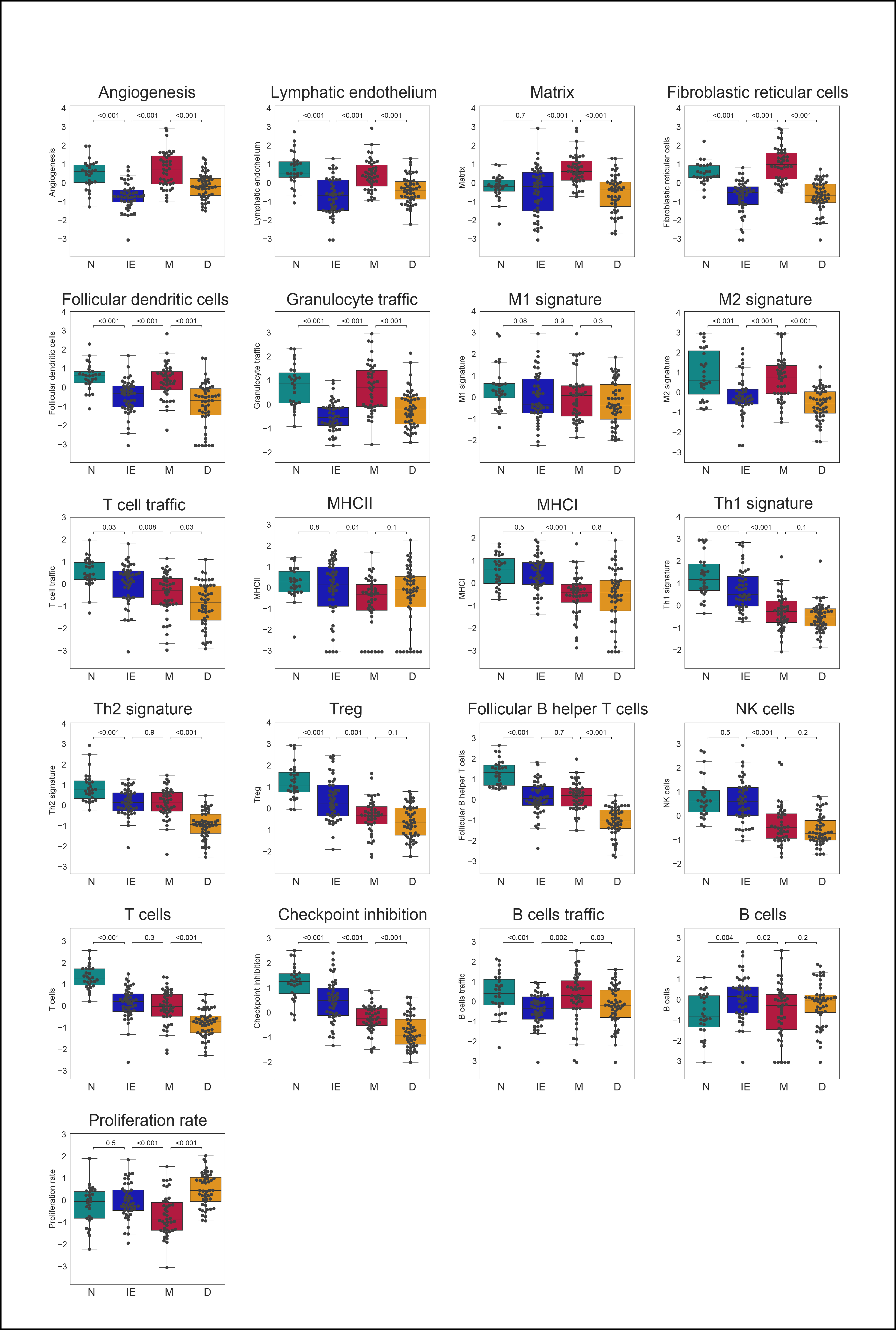

Supplement: Supplementary file 4 — Supplemental Figure-3 [file 41408_2023_927_MOESM4_ESM.tif]

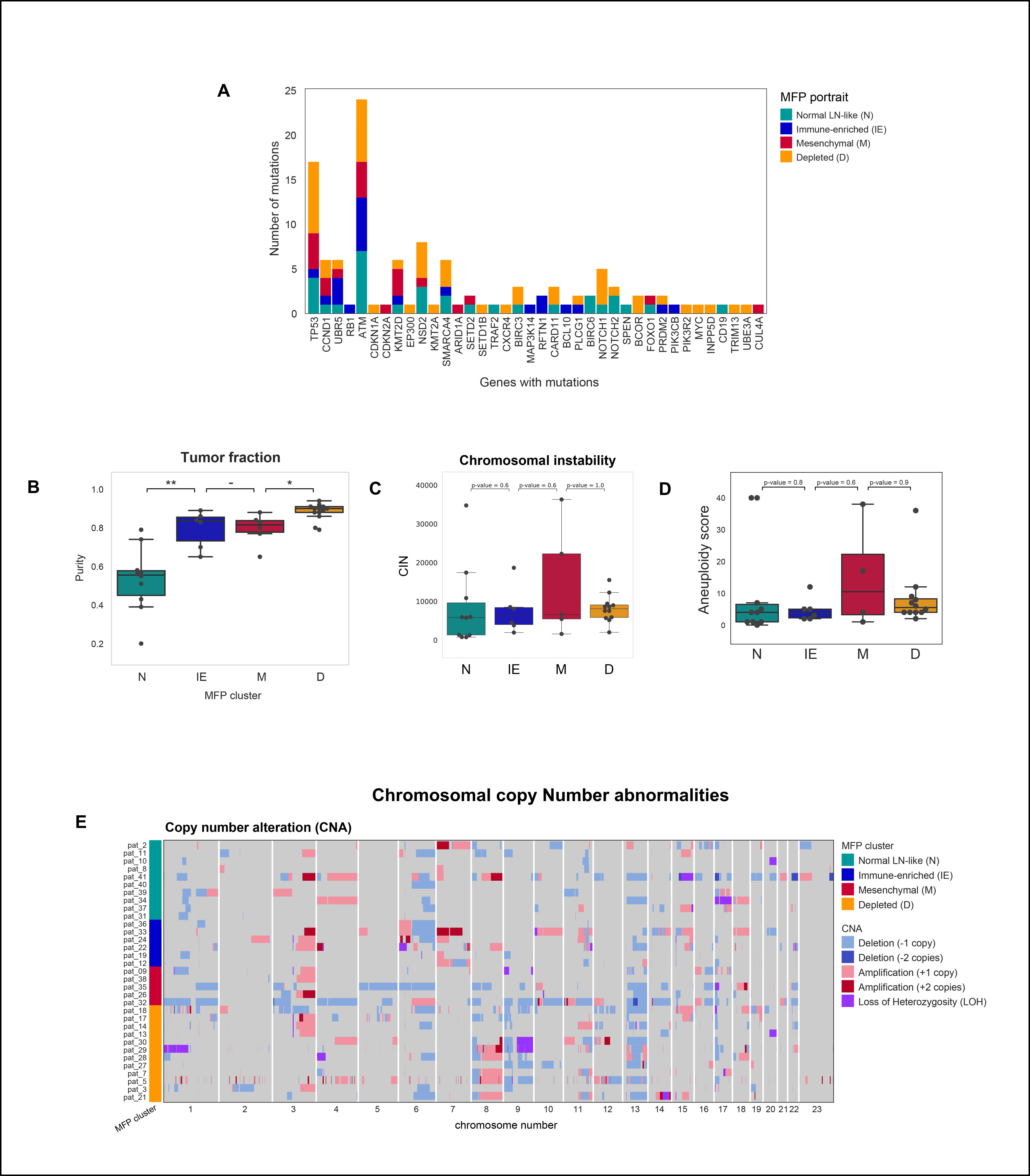

Supplement: Supplementary file 5 — Supplemental Figure-4 [file 41408_2023_927_MOESM5_ESM.tif]

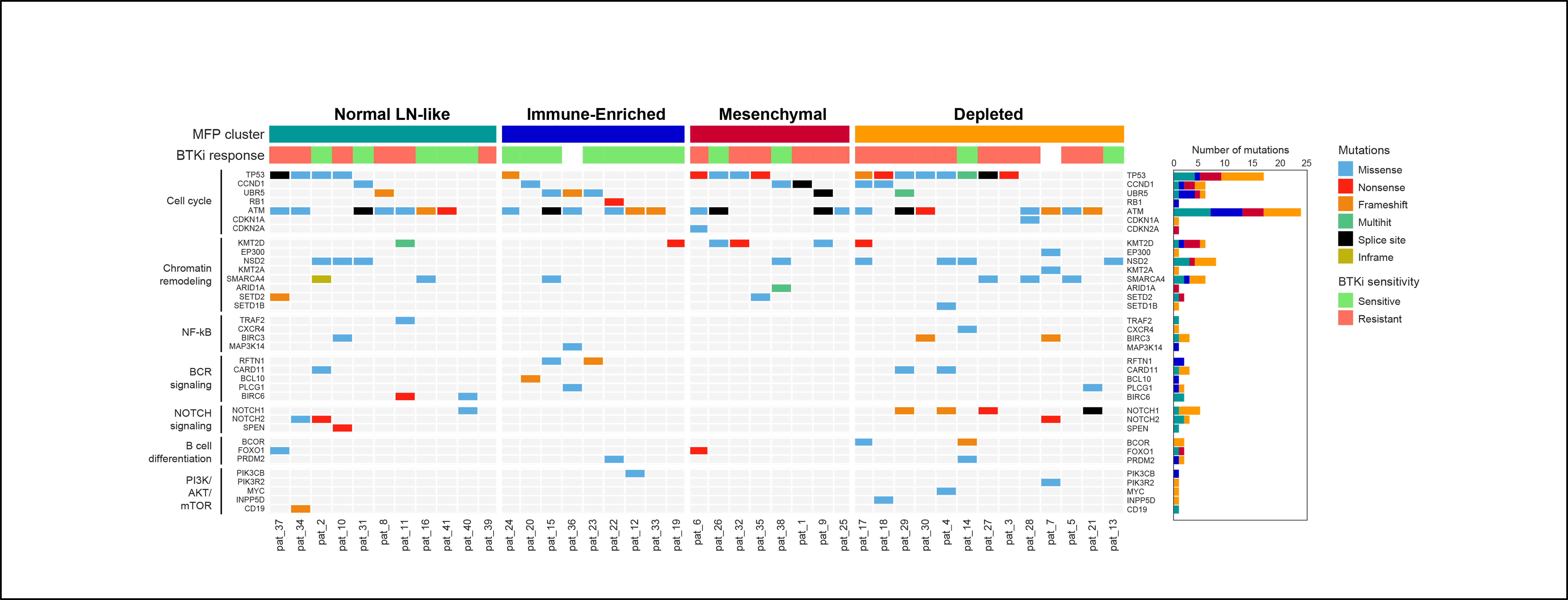

Supplement: Supplementary file 6 — Supplemental Figure-5 [file 41408_2023_927_MOESM6_ESM.tif]
