# Supplementary material for: Immune-depleted tumor microenvironment is associated with poor outcomes and BTK inhibitor resistance in mantle cell lymphoma
Source: Blood Cancer J. 2023 Oct 12;13(1):156. doi: 10.1038/s41408-023-00927-2 (PMC10567800; doi:10.1038/s41408-023-00927-2)
Supplement: Supplementary file 7 — Supplemental Table and figure legends [file 41408_2023_927_MOESM7_ESM.docx]

**Supplemental files**

**Supplemental figure Legends**

**Supplemental figure 1A:** Cell deconvolution for 41 samples with fixed annotation for all samples. Tissue detail line plot suggests more detailed information on Tissue site. Cell deconvolution for 41 MFP samples plus T2,T3,T4 samples derived from normal lymph nodes is shown. N cluster resembles normal LN samples: that explains where the name “normal lymph node-like” for this cluster is coming from. No variability of TME between tissue types was found except for fibroblasts, which is expected. Box plots for general LN and Tissue types – are also shown, no significant difference was found except for fibroblasts, which is expected.

**Supplemental figure 1B: A) TME types in MCL -** The TME is divided into immune enriched (N and IE subtype) and immune desert types (M and D subtypes). Most significant and specific gene signatures from figure 1B are shown, according to TME clusters. Favorable prognosis is shown in blue and adverse prognosis in red color blocks. Tumor proliferation rate signature is overexpressed in immune-depleted (D) TME cluster. **B)** **Box plots from selected PROGENy pathways.** Selected important PROGENy pathways from TP53, JAK-STAT, EGFR, NFkB, hypoxia, MAP kinase, Wnt and PI3Kinase pathways are shown according to the 4 Molecular Functional Portrait (MFP) clusters. Immune depleted (orange), mesenchymal (red), immune enriched (blue) and normal lymph node like TME cluster (green). PI3K pathway was overexpressed in immune depleted subtype.

**Supplemental figure 2: Box plots from immune chekpoint genes among the four TME (tumor microenvironment) clusters.** Main immune checkpoint genes (PDCD1, PD-L1, TIGIT, LAG3, and HAVCR3) expressions profile is shown according to the MFP cluster subtypes. Immune depleted (orange), mesenchymal (red), immune enriched (blue) and normal lymph node like TME cluster (green). The data is shown after z-score normalization. Immune depleted and mesenchymal subtypes had a lower expression of immune check point genes compared with immune rich and normal lymph node like MFP clusters.

**Supplemental figure 3: Box plots with gene signatures according to the four TME (tumor microenvironment) clusters in different cell types using a RNA sequencing deconvolution algorithm.** Immune depleted (orange), mesenchymal (red), immune enriched (blue) and normal lymph node like TME cluster (green). The proliferation rate gene signature is noticeably overexpressed in immune depleted subtype of TMP clusters.

**Supplemental figure 4: Somatic mutations according to the four TME (tumor microenvironment) clusters. A)** Oncoplot mutation distribution showing bar plots with distribution of highly mutated genes in mantle cell lymphoma, by molecular Functional Portrait (MFP) clusters. Immune depleted (orange), mesenchymal (red), immune enriched (blue) and normal lymph node like TME cluster (green). Multiple mutations were clustered in immune depleted subtype, as compared to other MFP subtypes. High risk somatic mutations *TP53, SMARCA4, NSD2, NOTCH1* were dominant in immune depleted subtype **B)** Tumor fraction purity showing box plots with % cellularity of mantle cell lymphoma (MCL) cells measured by variant allele frequency (VAF) in DNA samples according to the MFP subtype. Except the immune enriched (green) subtype, other subtypes had equivalent % cellularity. **C)** Chromosomal instability (CIN) is shown by box plots according to the MFP subtype. Immune depleted (D in orange) cluster, mesenchymal (M in red), immune enriched (B in blue) and normal lymph node like (A in green color). Both the immune desert subtypes, mesenchymal (red) and immune-depleted (orange) subtypes had a relatively higher Chromosomal instability **D)** Ploidy distribution shown by bar plots with di- or tetraploidy tumors by MFP subtype. 2N – diploid tumors and 4N – tetraploid tumors **E)** Copy number abnormalities (CNA) – CNA plots are shown with amplifications ( in red) and deletions ( in blue) of chromosomal regions per patient.

**Supplemental figure 5: Oncoplots with mutations within the four molecular functional portrait clusters (MFP) and arranged by functional genetic pathways and response to BTKi.** Immune depleted subtype (orange on top bar) showed maximum aberrancies in cell cycle and chromatin remodeling pathway with clusters of mutations in genes involved in these genetic pathways.

**Supplemental figure 6: Patterns of immunoglobulin heavy chain variable region genes (IGHV) in mantle cell lymphoma (MCL) and distribution according to the four molecular functional portrait (MFP) clusters. A)** The distribution of IGHV gene usage of the dominant (tumor) B cell receptor (BCR) clones in MCL patients. VH3-21, VH 3-23, VH 4-34, VH4-31, VH 4-59 and VH5-51 were predominant **B)** IGHV gene segment usage frequencies of the dominant (tumor) BCR clone and distribution according to BTKi sensitivity. VH3-21 (pink) and VH 3-23 (red) were dominant in ibrutinib resistant patient samples **C)** Stacked bar charts of the dominant (tumor) BCR clone and IGHV gene segment usage with number of patients within the 4 MFP clusters. VH3-21 (pink) and VH 3-23 (red) were noted in immune depleted (D) and mesenchymal (C) subtypes **D)** Box plots with the fraction (in %) of the tumor clone (measured by the counting of CDR3 amino acid sequence reads) in the detected BCR repertoire, distributed by MFP clusters.

**Methods**

This study was performed from retrospective patient tissue biopsy samples, collected after the provision of informed consent and approval by the Institutional Review Board at The University of Texas MD Anderson Cancer Center. A total of 41 patients were included. Among evaluable patients, DNA and RNA extraction was performed from fresh surgical biopsies from lymph nodes and non-nodal tissues from patients with relapsed MCL. Saliva samples were used for germline DNA. Whole exome (WES) and bulk RNA sequencing were performed to assess the somatic mutation profile, copy number abnormalities and gene expression profile to identify tumor microenvironment (TME) gene clusters.

**DNA and RNA Extraction**

For DNA and RNA sequencing, fresh specimens were immediately placed into RNALater solution after surgical biopsy or Ficoll-Hypaque density centrifugation and selective CD19 magnetic isolation of CD19^+^ cells. All procedures were performed in cold buffer or on ice. DNA and RNA extractions were performed at the MD Anderson Core Facility following standard protocols. Extractions using the QIAmp DNA kit (Qiagen) according to handbook protocol. DNA and RNA were checked for integrity and size distribution using the Agilent Bioanalyzer.

The majority of ibrutinib-resistant MCL samples were collected at the time or after progression on ibrutinib, and the sensitive samples were collected prior to ibrutinib therapy. Ficoll-Hypaque density centrifugation and anti-CD19 magnetic microbeads (Miltenyi Biotec) were used to isolate mononuclear cells.

**Whole-exome sequencing (WES)**

Briefly, indexed libraries were generated from 500 ng of sheared, genomic DNA (Bioruptor Ultrasonicator, Diagenode) using the KAPA Hyper Library Preparation Kit (Kapa Biosystems) and were prepared for capture with six cycles of preligation-mediated polymerase chain reaction (PCR) amplification. After amplification and reaction cleanup, the libraries were fluorometrically quantified with the Qubit dsDNA HS (High Sensitivity) Assay (Thermo Fisher Scientific) and analyzed for size distribution (Fragment Analyzer, Advanced Analytical). Normalization was performed, and the libraries were multiplexed at six libraries per pool. A probe pool was used to hybridize each multiplexed library pool with the SeqCap EZ Human Exome Enrichment Kit v3.0 (Roche NimbleGen). The enriched libraries were amplified with eight cycles of post-capture PCR and then examined for exon target enrichment by quantitative PCR (qPCR). The exon-enriched libraries were evaluated for size distribution (Fragment Analyzer, Advanced Analytical) and quantified by qPCR using the KAPA Library Quantification Kit (Kapa Biosystems). Sequencing was performed on the HiSeq 4000 Sequencer (Illumina), one capture (six samples) per lane using the 76–base pair (bp) paired-end configuration.

**Bulk RNA sequencing**

Illumina-compatible, barcoded, and strand-specific total RNA libraries were assembled with the TruSeq Stranded Total RNA Sample Preparation Kit (Illumina). Cytoplasmic and mitochondrial ribosomal RNAs were removed from 250 ng of deoxyribonuclease I–treated total RNA with Ribo-Zero Gold (Illumina). Divalent cations were used to fragment the RNA after purification, and double-stranded complementary DNA (cDNA) was synthesized. After synthesis and repair, Illumina-specific indexed adapters were ligated. Purification and enrichment with 12 cycles of PCR were conducted to prepare the cDNA library. Each library was then quantified using the Qubit dsDNA HS Assay (Thermo Fisher Scientific) and multiplexed into pools containing 24 libraries. Pooled libraries were quantified using the KAPA Library Quantification Kit (Kapa Biosystems), examined for size distribution using the Fragment Analyzer (Advanced Analytical), and then, using the 76-bp paired-end format, sequenced in four lanes of the Illumina HiSeq 4000 Sequencer *(further details on methods and analysis are in supplemental file)*.

**Quality control**

Raw FASTQ files were analysed with FastQC (v0.11.5), Fastq-Screen (v0.14), mosdepth (v0.3.1), RSeQC (v1.1.8). RNAseq files considered passed in case <2% contamination with other species, >10M protein-coding reads, no evidence of RNA degradation by RSEqc. WES files considered passed in case >50X coverage per nucleotide, <2% contamination, <30% duplicates. All samples passed quality control for this research.

## **DNA-seq Pipeline**

**Data Alignment**

Low quality reads were filtered using FilterByTile/BBMap v37.90, others were aligned to the GRCh38 (GRCh38.d1.vd1 assembly) human reference genome using BWA v0.7.17. Duplicate reads were marked using Picard’s v2.20.7 MarkDuplicates; indels are realigned and further recalibrated by BaseRecalibrator (GATK v4.1.2.0). Tumor and germline samples underwent the same procedures. Both germline and somatic variant calling was performed using Strelka v2.9.10 with MANTA v1.6.0. Copy number alterations were detected using Sequenza.

**RNA-seq Pipeline**

RNA-seq reads were processed using a unified pipeline (Vivian J, Rao AA, Nothaft FA, Ketchum C, Armstrong J, Novak A, et al. Toil enables reproducible, open source, big biomedical data analyses. (*Nat Biotechnol 2017;35:314–6*). Reads were aligned using Kallisto v0.42.4 to GENCODE v23 transcripts 69 with default parameters. Then, protein coding, IGH/K/L- and TCR-related transcripts were retained, whereas noncoding, histone- and mitochondrial-related transcripts were removed, resulting in 20,062 analyzed transcripts. Gene expression was quantified as transcripts per million and log2 transformed.

**Tumor Microenvironment Reconstruction (Deconvolution)**

BostonGene utilizes a proprietary ML-based algorithm named Kassandra (DOI: 10.1158/1538-7445.AM2020-853) which provides a robust tool for cell deconvolution. From bulk RNA-sequencing mix Kassandra quantifies the cell type composition enabling remodeling of a patient-specific tumor microenvironment.

Kassandra is based on LightBGM (v 2.3.1 or later) model trained on artificial RNA-sequencing mixes mimicking the cellular complexity and diversity of tumor biopsies, which enables the algorithm to accurately identify closely related cell subpopulations with highly similar expression patterns.

Cell ratios are calculated according to the formula:

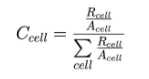


Where C_cell_ is cell fraction of cell type, R_cell_ is RNA fraction of cell type, A_cell_ is relative RNA per cell coefficient and cellR_cell_=1.

Cell types or subtypes that included or were a subset of the used types were recalculated according to the change in used types.

**MFP TME identification**

The TME signatures were curated from the previous study in diffuse large B cell lymphoma (10.1158/2159-8290.CD-20-0839). Functional gene expression signatures (F-GES) of tumor microenvironmental cells, cellular states, physiological and pathological processes and signaling pathways were developed using a combination of gene expression signatures and literature curation. F-GES for cell types were obtained from purified gene expression cell population signatures manually curated to include only those genes that are exclusively expressed in the defined cell type or specifically associated with a particular biological process using data from more than 500 publications.

Signature scores were calculated using in-house python implementation of the ssGSEA. F-GES intensities were median-transformed in MDA and Scott et al. cohorts separately and then combined. TME clustering: F GES signatures were used to identify microenvironmental patterns among MCL gene expression samples by unsupervised dense clustering using the Louvain method for community detection. Inter-sample similarity was calculated using Pearson correlation. The resulting distance matrix was converted into a graph where each sample formed a node and two nodes formed an edge with weight equal to the pair’s Pearson correlation. Edges with weight lower than specified threshold (0.45) were removed and the Louvain community detection algorithm was applied to calculate graph partitioning into clusters. To mathematically determine the optimum threshold for observed clusters we employed minimum David Bolduin, maximum Calinski Harabasz and Silhouette scores excluding separations with low-populated clusters (< 5% of samples).

**B-cell Receptor Repertoire Assessment**

To process immunome data from RNA sequences into quantitated clonotypes, we applied MiXCR v2.1.7 (Bolotin DA, Poslavsky S, Davydov AN, Frenkel FE, Fanchi L, Zolotareva OI, et al. Antigen receptor repertoire profiling from RNA-seq data. Nat Biotechnol 2017;35:908–11.). Single clonotypes were grouped into clones with unique VDJ combination and identical CDR3 nucleotide sequences. For B cells, the clones were further aggregated into clone groups if the VDJ combination was the same and CDR3 nucleotide sequences differed no more than 1 nucleotide. The biggest clone group was assigned as tumor if the absolute clonotype counts > 20; the relative clonotype counts > 5%; the ratio of the second biggest group to the first < 0.6 and the group contains an enriched clone > 25%. The tumor light chain was called if there was an enriched clonotype in one of the light chains. In cases with an enriched clone in both chains, the biggest by absolute counts was selected.

**RNA mutation calling**

Mutation calling from RNASeq was performed using strelka germline. Then potential germline mutations were filtered according to max allele frequency in the human population and variant allele frequency in the data.

**References -**

1. Li H and Durbin R. Fast and accurate short read alignment with Burrows-Wheeler transform. *Bioinformatics* 2009; 25: 1754-1760. DOI: 10.1093/bioinformatics/btp324.

2. DePristo MA, Banks E, Poplin R, et al. A framework for variation discovery and genotyping using next-generation DNA sequencing data. *Nat Genet* 2011; 43: 491-498. DOI: 10.1038/ng.806.

3. Rimmer A, Phan H, Mathieson I, et al. Integrating mapping-, assembly- and haplotype-based approaches for calling variants in clinical sequencing applications. *Nat Genet* 2014; 46: 912-918. DOI: 10.1038/ng.3036.

4. Cibulskis K, Lawrence MS, Carter SL, et al. Sensitive detection of somatic point mutations in impure and heterogeneous cancer samples. *Nat Biotechnol* 2013; 31: 213-219. DOI: 10.1038/nbt.2514.

5. Ye K, Schulz MH, Long Q, et al. Pindel: a pattern growth approach to detect break points of large deletions and medium sized insertions from paired-end short reads. *Bioinformatics* 2009; 25: 2865-2871. DOI: 10.1093/bioinformatics/btp394.

6. Liu X, Wu C, Li C, et al. dbNSFP v3.0: A One-Stop Database of Functional Predictions and Annotations for Human Nonsynonymous and Splice-Site SNVs. *Hum Mutat* 2016; 37: 235-241. DOI: 10.1002/humu.22932.

7. Adzhubei I, Jordan DM and Sunyaev SR. Predicting functional effect of human missense mutations using PolyPhen-2. *Curr Protoc Hum Genet* 2013; Chapter 7: Unit7 20. DOI: 10.1002/0471142905.hg0720s76.

8. Kumar P, Henikoff S and Ng PC. Predicting the effects of coding non-synonymous variants on protein function using the SIFT algorithm. *Nat Protoc* 2009; 4: 1073-1081. DOI: 10.1038/nprot.2009.86.

9. Schwarz JM, Cooper DN, Schuelke M, et al. MutationTaster2: mutation prediction for the deep-sequencing age. *Nat Methods* 2014; 11: 361-362. DOI: 10.1038/nmeth.2890.

10. Reva B, Antipin Y and Sander C. Predicting the functional impact of protein mutations: application to cancer genomics. *Nucleic Acids Res* 2011; 39: e118. DOI: 10.1093/nar/gkr407.

11. Chun S and Fay JC. Identification of deleterious mutations within three human genomes. *Genome Res* 2009; 19: 1553-1561. DOI: 10.1101/gr.092619.109.

12. Shihab HA, Rogers MF, Gough J, et al. An integrative approach to predicting the functional effects of non-coding and coding sequence variation. *Bioinformatics* 2015; 31: 1536-1543. DOI: 10.1093/bioinformatics/btv009.

13. Quang D, Chen Y and Xie X. DANN: a deep learning approach for annotating the pathogenicity of genetic variants. *Bioinformatics* 2015; 31: 761-763. DOI: 10.1093/bioinformatics/btu703.

14. Choi Y, Sims GE, Murphy S, et al. Predicting the functional effect of amino acid substitutions and indels. *PLoS One* 2012; 7: e46688. DOI: 10.1371/journal.pone.0046688.

15. Carter H, Douville C, Stenson PD, et al. Identifying Mendelian disease genes with the variant effect scoring tool. *BMC Genomics* 2013; 14 Suppl 3: S3. DOI: 10.1186/1471-2164-14-S3-S3.

16. Kircher M, Witten DM, Jain P, et al. A general framework for estimating the relative pathogenicity of human genetic variants. *Nat Genet* 2014; 46: 310-315. DOI: 10.1038/ng.2892.

17. Davydov EV, Goode DL, Sirota M, et al. Identifying a high fraction of the human genome to be under selective constraint using GERP++. *PLoS Comput Biol* 2010; 6: e1001025. DOI: 10.1371/journal.pcbi.1001025.

18. Dong C, Wei P, Jian X, et al. Comparison and integration of deleteriousness prediction methods for nonsynonymous SNVs in whole exome sequencing studies. *Hum Mol Genet* 2015; 24: 2125-2137. DOI: 10.1093/hmg/ddu733.

19. Zhang J, Fujimoto J, Zhang J, et al. Intratumor heterogeneity in localized lung adenocarcinomas delineated by multiregion sequencing. *Science* 2014; 346: 256-259. DOI: 10.1126/science.1256930.

20. Olshen AB, Venkatraman ES, Lucito R, et al. Circular binary segmentation for the analysis of array-based DNA copy number data. *Biostatistics* 2004; 5: 557-572. DOI: 10.1093/biostatistics/kxh008.

**Supplemental Table-1 Baseline patient characteristics (n=41)**

| **Pat code** | **Age at treatment start** | **Gender** | **Ki-67% in tissue** | **Tissue Type** | **OS (Days)** | **BTKi_response** | **Treatments** |
| --- | --- | --- | --- | --- | --- | --- | --- |
| Pat-01 | 69 | M | 90 | Mediastinal  LN | 262 | Acquired Resistance | Dex; R-CHOP×4; R-DHAP×2, carfilzomib-ibrutinib progression |
| Pat-02 | 83 | F | 70 | LN | 335.0 | Sensitive | R-CVPx6 with maintenance Rituxan (2006), BR x4 (2016), IR (10/2016) and radiation (stopped ibrutinib 3/2017 due to rash and a-fib), Len-rituximab, Len |
| Pat-03 | 72 | M | 85 | LN | 557.0 | Primary Resistance | BR-bortezomib, R-HCVAD, Zanubrutinib, ibrutinib progression in 1 cycle then Bortezomib, len rituximab, dexamethasone |
| Pat-04 | 73 | M | 55 | LN | 1109.0 | Primary Resistance | R-CHOP alternating with Cytarabine (Nordic) + SCT, relapse (11/2015), IR+ radiation, anti-CD19 CAR-T cells |
| Pat-05 | 71 | M | 60 | LN | 104.0 | Primary Resistance | R-CHOP x6, SCT (1/2012) + maintenance Rituxan (until 2014), BRx6 (2015), Ibrutinib, R-HyperCytoxan-dexamethasone + radiation, Fludarabine cyclophosphamide, and intrathecal chemotherapy for CNS disease (5/27/2016) |
| Pat-06 | 76 | F | 90 | Soft Tissue | 1119.0 | Acquired Resistance | Rituximab, BRx4, R-Hyper-CVAD + IT chemo one cycle, IR, SCT, Ofa + Ibrutinib, Len-rituximab, bortezomib with Dexamethasone, DLI (7/2016), Ibrutinib (9/2017) then switched to Venetoclax + radiation(10/2017) |
| Pat-07 | 63 | M | 65 | LN | 168.0 | NA | R-HCVAD then BR then zanubrutinib for 10 days then stopped due to infections |
| Pat-08 | 57 | M | 75 | LN | 1029.0 | Acquired Resistance | Bortezomib-R-HCVAD then acalabrutinib then progression then carfilzomib-ibrutinib then anti-CD19 CART |
| Pat-9 | 71 | M | 90 | LN | 860.0 | Acquired Resistance | Bortezomib-R-HCVAD then IR then splenectomy then anti-CD19 CART |
| Pat-10 | 79 | M | 15 | LN | 334.0 | Acquired Resistance | Radiation then IR |
| Pat-11 | 67 | M | 40 | LN | 791.0 | Primary Resistance | Bortezomib + R-hyperCVAD then Ibrutinib then anti CD19 CART-T infusion |
| Pat-12 | 59 | M | 65 | LN | 894.0 | Sensitive | R-hyperCVAD × 4; R-EPOCH × 4; R × 4; Bortezomib × 4; R-Lenalidomide; R-Bendamustine × 1; Temsirolimus; Methotrexate and cytarabine × 1,bortezomib then ibrutinib discontinued due to bleeding in pituitary and started carfilzomib |
| Pat-13 | 76 | M | NA | LN | 536.0 | Sensitive | R-hyperCVAD × 6; R-bortezomib and cyclophosphomide × 6, IR, discontinued ibrutinib due to bleeding |
| Pat-14 | 48 | M | 60 | LN | NA | Sensitive | R-HCVAD then bortezomib then BR then Radiation then len-rituximab then IR then SCT then PD |
| Pat-15 | 67 | M | 55 | LN |  | Sensitive | Bortezomib-R-HCVAD then IR then secondary AML |
| Pat-16 | 73 | M | 5 | LN | 2057.0 | Sensitive | R-HCVAD × 6; Bortezomib × 4, IR continued |
| Pat-17 | 69 | F | 95 | Soft Tissue | 176.0 | Acquired Resistance | R-HCVAD × 2; Rituximab × 8; Len-rituximab; Bortezomib × 4 then IR with subsequent PD |
| Pat-18 | 67 | M | 90 | LN | 127.0 | Acquired Resistance | R-hyperCVAD with methotrexate and cytarabine plus bortezomib × 7; Bortezomib × 1 then single agent ibrutinib |
| Pat-19 | 72 | M | 20 | LN | 2004.0 | Sensitive | R-CHOP × 6; Rituximab × 8 then IR later discontinued due to atrial fibrillation then len-rituxumab |
| Pat-20 | 66 | F | 30 | LN | 2047.0 | Sensitive | R-CHOP × 4 then IR then discontinued with infections then CART therapy |
| Pat-21 | 70 | M | 60 | LN | 414.0 | Primary Resistance | R-hyperCVAD with methotrexate and cytarabine × 6, bortezomib then splenectomy then IR then carfilzomib, len rituximab |
| Pat-22 | 59 | M |  | LN | 1807.0 | Sensitive | R-CHOP × 6; R-HCVAD alternating with R-methotrexate and cytarabline × 1; Rituximab x 24; BR then IR |
| Pat-23 | 79 | M | 35 | Salivary gland | 1216.0 | Sensitive | R-CHOP with Bortezomib × 6; R-bendamustine × 4; Radiation therapy; Lenalidomide × 2; R-cladribine and cyclophosphomide × 1 then IR withdrew consent due to side effects then bortezomib |
| Pat-24 | 88 | M | 45 | Soft Tissue | 905.0 | Sensitive | R-CHOP x 6; BR x 4; R-bortezomib and dexamethasone x2; Bendamustine then IR and discontinued for adverse events then single agent ibrutinib |
| Pat-25 | 74 | F | 90 | Soft Tissue | 89.0 | Primary Resistance | BR x 3 then IR then progressed then bortezomib, Len-rituximab then R-DHAP |
| Pat-26 | 77 | M | 30 | Soft Tissue | 469.0 | Sensitive | BR x6 (4/2016), Acalabrutinib-obinutuzmab |
| Pat-27 | 61 | M | 75 | LN | 26.0 | Acquired Resistance | IR then R-HCVAD then progression |
| Pat-28 | 62 | M |  | Soft Tissue | 2 yrs | Acquired Resistance | BR, Ibrutinib, len, auto-SCT, venetoclax, R-hypercytoxan dexa, acalabrutinib then held due to CMV retinitis |
| Pat-29 | 62 | M | 85 | Soft Tissue | 540.0 | Primary Resistance | Maxi-CHOP per Nordic regimen x6 (1/12/2017), auto-SCT-rituximab, Ibrutinib (11/2017), R-hyperCVAD (11/20/2017), DR2iVe- Velcade + Rituxan + Revlimid + Dexamethasone (12/13/2017), then anti-CD19 CART then later secondary AML |
| Pat-30 | 68 | F | 90 | LN | 20.0 | Primary Resistance | Vorinostat cladribine rituximab then bortezomib-rituximab, BR, acalabrutinib then progression, ibrutinib then progression then bortezomib-rituximab--len-dexa, venetoclax |
| Pat-31 | 73 | F | 10 | LN | 317.0 | Sensitive | BR then acalabrutinib |
| Pat-32 | 73 | M | 70 | Tissue-gastric | 227.0 | Acquired Resistance | CHOP x8, R-hyper-CVAD x6, Bortezomib x2, Bortezomib-BR, IR, Bortezomib-Len-rituximab, Venetoclax, R-MINE |
| Pat-33 | 75 | M | 45 | LN | 249.0 | Sensitive | BR x6 (1/2015), maintenance Rituxan for 1 year, low-dose radiation, Ibrutinib and Venetoclax |
| Pat-34 | 73 | F | 40 | LN | 378.0 | Primary Resistance | BR then Ibrutinib x6, continued maintenance Ibrutinib then Len-rituximab |
| Pat-35 | 54 | F | 70 | Bone-rib | 314.0 | Acquired Resistance | R-CHOP alternating R-DHAP x6 followed by BEAM with auto-SCT, Acalabrutinib then progressed then Radiation with Len-rituximab |
| Pat-36 | 73 | M | 35 | LN | 9.0 | NA | Treated outside with acalabrutinib |
| Pat-37 | 70 | M | 70 | LN | 331.0 | Primary Resistance | BR x6 (9/2014), acalabrutinib, IR, R-Hypercyclophosphamide-dexax3, Radiation, Bortezomib-len-rituximab |
| Pat-38 | 58 | M | 35 | LN | 223.0 | Sensitive | Bortezomib-R-HCVAD, IR then discontinued due to side effects |
| Pat-39 | 53 | M | 20 | Soft Tissue | 238.0 | Acquired Resistance | IR-R-HCVAD then progressed later acalabrutinib-obinutuzumab |
| Pat-40 | 73 | M | 5 | LN | 889.0 | Sensitive | IR |
| Pat-41 | 91 | F | 50 | LN | 570.0 | Primary Resistance | Single agent rituximab, Ibrutinib-obinutuzumab, BR |
